# Exploring open science in applied ethology: practice and attitudes among researchers

**DOI:** 10.1186/s13104-026-07685-x

**Published:** 2026-02-06

**Authors:** Christian Nawroth, Helen Gray, I. Anna S. Olsson

**Affiliations:** 1 Research Institute for Farm Animal Biology (FBN), Dummerstorf, Germany; 2 School of Natural and Environmental Sciences, Newcastle University, Newcastle upon Tyne, UK; 3 i3S - Instituto de Investigação e Inovação em Saúde, Universidade do Porto, Porto, Portugal

**Keywords:** Open data, Preprint, Preregistration, Survey

## Abstract

**Objective:**

In light of the replication crisis, transparency is critical for impactful research. This is particularly relevant for animal-based research fields, like Applied Ethology, where ethical obligations demand both scientific integrity and responsible use of animals. While Open Science practices are well-established in some fields, especially psychology, they remain limited in the field of Applied Ethology, for which there is also a lack of standardised guidelines and training. To assess the prevalence of different Open Science practices in this field, we surveyed attendees of the 2021 virtual International Society for Applied Ethology Congress.

**Results:**

Of 372 invitations, 112 surveys were completed (30.1%). Preprints (25%) and preregistration (9%) were uncommon, with lack of training cited as the main barrier. Of those who had published a preprint/preregistered study protocol, preprints were mainly used for result dissemination (81%) and preregistration for quality control and integrity (56%). 27% reported always sharing their data, 57% shared sometimes, and 15% never shared their data. Sharing data as supplementary material was most common, only a minority used data repositories and only 21% of respondents had heard of the FAIR principles.

**Supplementary Information:**

The online version contains supplementary material available at 10.1186/s13104-026-07685-x.

## Introduction

Open Science is a transformative movement that aims to improve the transparency, accessibility and reproducibility of scientific research through practices such as Open Data, Open Access publishing and collaborative research approaches [1]. By openly sharing data, methods and materials, researchers can help address the reproducibility crisis in science by promoting transparency and accountability. Preregistration reduces publication bias and researcher degrees of freedom by ensuring that research hypotheses and methods are defined in advance [2], while preprinting and Open Access publishing boost visibility and impact [3]. Collaborative research practices can promote greater rigour and inclusivity in scientific investigations, making Open Science a powerful framework for producing reliable and robust scientific knowledge [4, 5].

Open Science is especially valuable in animal research, where ethical and scientific integrity are crucial. Ethical guidelines, including the 3Rs principle [6], emphasize minimizing animal use and ensuring humane treatment. Open data sharing helps reduce animal use by preventing redundant studies [7] while improving research quality and robustness. Robust and transparent studies are particularly critical when the findings can influence policy or guidelines, such as in Applied Ethology, where research often leads to recommendations on practices such as husbandry and routine procedures [8]. Given the global impact on billions of animals, ensuring open and rigorous research is critical.

Open science is having a significant impact in various research fields. In psychology, preprint-friendly policies, Registered Reports, and Open Data repositories have been adopted by journals [9]. However, Applied Ethology lacks systematic guidelines and initiatives in this regard, with no available data looking at the prevalence of OS practices in this research field yet. An understanding of the current prevalence of Open Science practices and the perceived barriers to their implementation is important to design future Open Science initiatives to meet the specific needs of Applied Ethologists. To determine the extent to which Open Science practices are being adopted in the field, a survey was distributed to all attendees of the International Society for Applied Ethology (ISAE) 2021 International Congress, which was conducted online due to COVID-19 restrictions. The survey aimed to assess participants’ knowledge, implementation, and perceived benefits and barriers associated with Open Science practices in their research field. The survey focused primarily on three key implementations: preprints, preregistration of experimental protocols, and making data publicly available.

## Methods

We invited all registered participants of the International Congress of the International Society for Applied Ethology (ISAE), which took place from 02.08.2021–06.08.2021, in a virtual setting. A total of 372 emails were sent using the congress organiser’s web-based registration system. Emails were sent on 27 July 2021 and again on 30 July 2021. The survey closed on 1 August 2021. Of the 372 emails sent, 112 completed surveys were returned (response rate = 30.1%). The respondents had to complete a questionnaire hosted on EUSurvey (https://ec.europa.eu/eusurvey/home/welcome*).* All questions can be found in Table 1.

**Table 1:** Individual survey questions

Topic	Question	Response category	Response type and options
Demographics	Are you or have you been involved in research?	Single choice	“Yes, right now” “Yes, in the past” “No”
	Are you a member of the International Society for Applied Ethology (ISAE)?	Single choice	“Yes” “No”
	What is your educational status?	Single choice	“no PhD and not enrolled in PhD program” “enrolled in PhD program but PhD not completed yet” “PhD completed no more than 5 years ago” “PhD completed 5 or more years”
	How old are you?	Single choice	“18–30” “31–45” “46–60” “61 and above”
Pre-printing	Have you published a pre-print?	Single choice	“Yes” “No”
	Why have you not yet published a pre-print?	Multiple choice	“There is nothing in it for me” (lack of incentives) “My colleagues/coauthors/supervisors do not support this” (lack of support) “I simply don’t have the time for this” (lack of time) “I do not know how to do it” (lack of training) “I worry that others will steal my work” (fear of plagiarism)
	Please include here anything specific that has prevented you from publishing a pre-print.	Free text response	
	Why have you published a pre-print?	Multiple choice	“Others can have early access to my work” (dissemination) “I can get early credit for my work” (acknowledgement) “Some results (e.g. negative results) are harder to publish, and I want to make all of my work available (transparency) “Although the peer-reviewed paper will be behind a paywall, everyone can download the pre-print” (accessibility)
	Please include here anything specific that facilitated your process of publishing a pre-print.	Free text response	
	How often did you publish a pre-print?	Single choice	“Once” “2–3 times” “More than 3 times”
	Where did you publish most of your pre-prints?	Single choice	“BioRxiv” “Open Science Framework (OSF)” “Other”
	Why do you think other researchers do not publish pre-prints?	Multiple choice	“There is nothing in it for them” (lack of incentives) “Their colleagues/coauthors/supervisors do not support this” (lack of support) “They simply don’t have the time for this” (lack of time) “They do not know how to do it” (lack of training)“ “I worry that others will steal my work” (fear of plagiarism)
	Please include here anything specific that may have prevented them from publishing a pre-print.	Free text response	
	Do you plan to publish a pre-print in the future?	Single choice	“Yes” “No” “I don’t know”
	How useful do you think a wider use of pre-prints would be for animal welfare science?	Scale	1 = not useful, 5 = very useful, leave blank for “I don’t know”
	Please explain your answer	Free text response	
Preregistration	Have you preregistered a study before?	Single choice	“Yes” “No”
	Why have you not preregistered a study yet?	Multiple choice	“There is nothing in it for me” (lack of incentives) “My colleagues/coauthors/supervisors do not support this” (lack of support) “I simply don’t have the time for this” (lack of time) “I do not know how to do it” (lack of training)
	Please include here anything specific that prevented you from preregistering a study.	Free text response	
	Why have you preregistered a study?	Multiple choice	“I can get expert-feedback via registered reports on my study design” (external quality control) “It improves the quality of my work if I must ensure that a very detailed test protocol and analysis plan are in place before I start data collection” (internal quality control) “I will have a time-stamp on my test protocol and ensure I’m credited for it” (anti-scooping) “It helps to avoid questionable research practices/researcher degrees of freedom and thus help to make my work more replicable” (integrity)
	Please include here anything specific that lead you to preregister a study.	Free text response	
	How often did you preregister a study?	Single choice	“Once” “2–3 times” “More than 3 times”
	Where did you pre-register your research?	Single choice	“Open Science Framework (OSF)” “As a Registered Report with a journal” “Other”
	Why do you think other researchers do not preregister their studies?	Multiple choice	“There is nothing in it for them” (lack of incentives) “Their colleagues/coauthors/supervisors do not support this” (lack of support) “They simply don’t have the time for this” (lack of time) “They do not know how to do it” (lack of training)
	Please include here anything specific that may have prevented them from preregistering a study.	Free text response	
	Do you plan to preregister a study in the future?	Single choice	“Yes” “No” “I don’t know”
	How useful do you think a wider use of preregistrations would be for animal welfare science?	Scale	1 = not useful, 5 = very useful, leave blank for “I don’t know”
	Please explain your answer	Free text response	
Open data	Do you make your research data publicly available so that others can access it without contacting you?	Single choice	“Never” “Sometimes” “Always”
	Which is the main way that you make data available?	Single choice	“As supplementary material to publications” “In a data repository” “Other (please specify)”
	If other, please specify	Free text response	
	Would you be interested in accessing other researchers’ data?	Single choice	“Yes, for transparency” “Yes, to use for research purposes” “No” “I don’t know”
	Do you know about the FAIR principles?	Single choice	“Yes” “No”

### Demographics

In an initial section, respondents were asked about their current involvement in research, ISAE membership status, educational status, and age group.

### Preprint

This section contained eight questions (including additional free text options to elaborate on answers) about the prevalence of and attitudes towards preprinting practices. Question 1 asked whether respondents had ever preprinted (yes/no). Questions 2–3 asked respondents to explain why they had or had not yet published a preprint. For those who answered ‘yes’ to having preprinted, questions 4 and 5 asked about the frequency and repositories used to publish preprints. Question 6 aimed to identify the attitudes that respondents perceived their colleagues to have towards the reluctance to publish preprints. Question 7 asked whether respondents expected to publish preprints in the future. Finally, question 8 asked about the potential impact of the wider use of preprints on animal welfare science.

### Preregistration

This section contained eight questions (including additional free text options to elaborate on answers) about the prevalence of and attitudes towards the preregistration of research protocols. Question 1 asked whether respondents had ever preregistered protocols (yes/no). Questions 2 and 3 asked respondents to explain why they had or had not preregistered protocols. For those who answered ‘yes’ to preregistration, questions 4 and 5 asked about the frequency and platforms/formats used to submit these preregistrations. Question 6 aimed to identify the attitudes that researchers believe their colleagues have towards the reluctance to preregister protocols. Question 7 asked whether respondents expected to submit preregistrations in the future. Finally, question 8 asked about the potential impact of the wider use of preregistrations on animal welfare science.

### Open data

This section included four questions (with additional free text options to elaborate on answers) about the prevalence of and attitudes towards making research data publicly available. Question 1 asked whether researchers routinely make their research data publicly available (always/sometimes/never). Question 2 asked about the platforms/formats used for making data publicly available. Question 3 asked respondents to elaborate on whether and why they would be interested in accessing other researchers’ data. Finally, question 4 asked respondents if they were familiar with the FAIR principles (yes/no; [17]).

## Results and discussion

The final dataset comprised 112 responses. Most respondents were actively involved in research, with a spread of career stages and ages (Table S1). To ensure consistency, responses containing contradictory information were excluded. For instance, if a respondent indicated they had not published a preprint but then specified a platform where they had done so, their data was removed from consideration. Prevalence and categorisation of free-text responses can be found in the ESM (S3 and S4).

### Preprints and preregistration

**Fig. 1:**
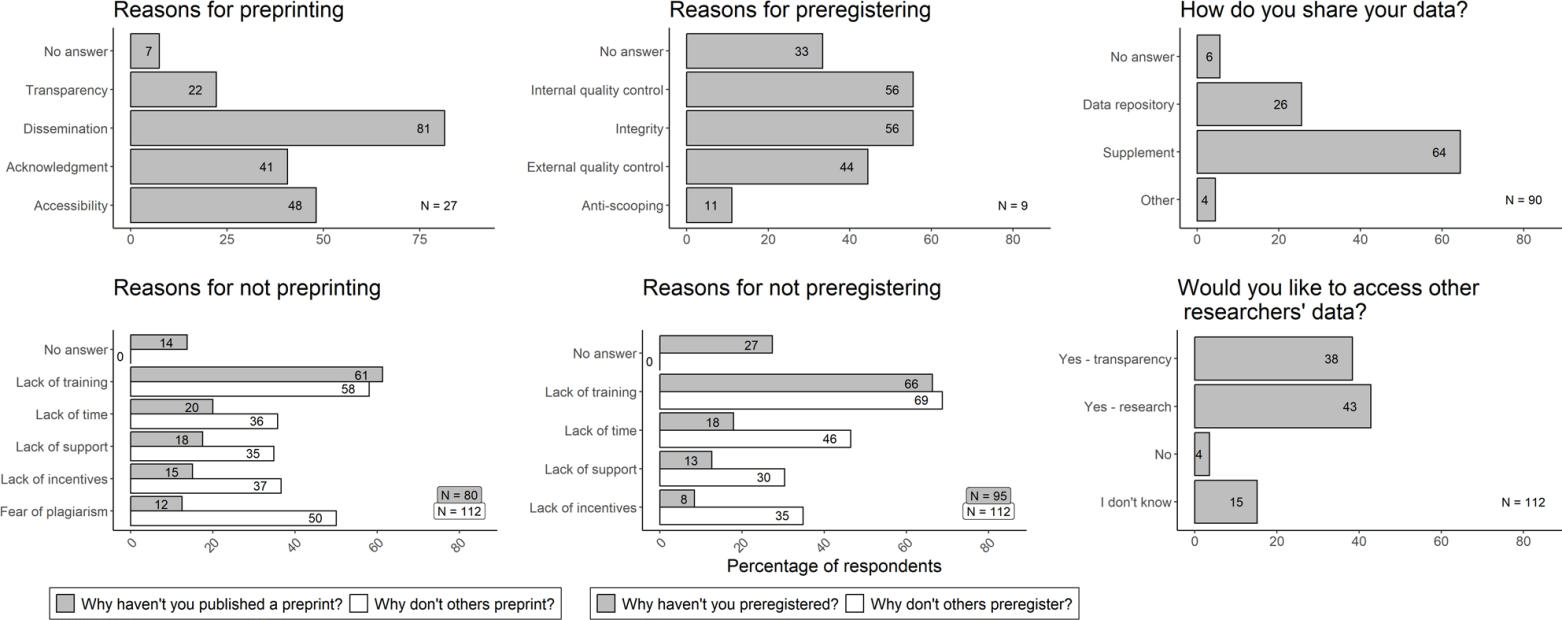
Respondents’ selections from predefined lists of reasons for: publishing a preprint (top left); having not published a preprint and their perceptions of why others had not published preprints (bottom left); preregistering (top middle); having not preregistered and their perceptions of why others had not preregistered (bottom middle). The percentage of respondents who deposit data in different ways (top right) and whether and why researchers are interested in accessing other researchers’ data (bottom right). Numbers on the bars refer to percentages of respondents. Note that multiple choices per respondent were possible and numbers refer to percentages of respondents

**Fig. 2:**
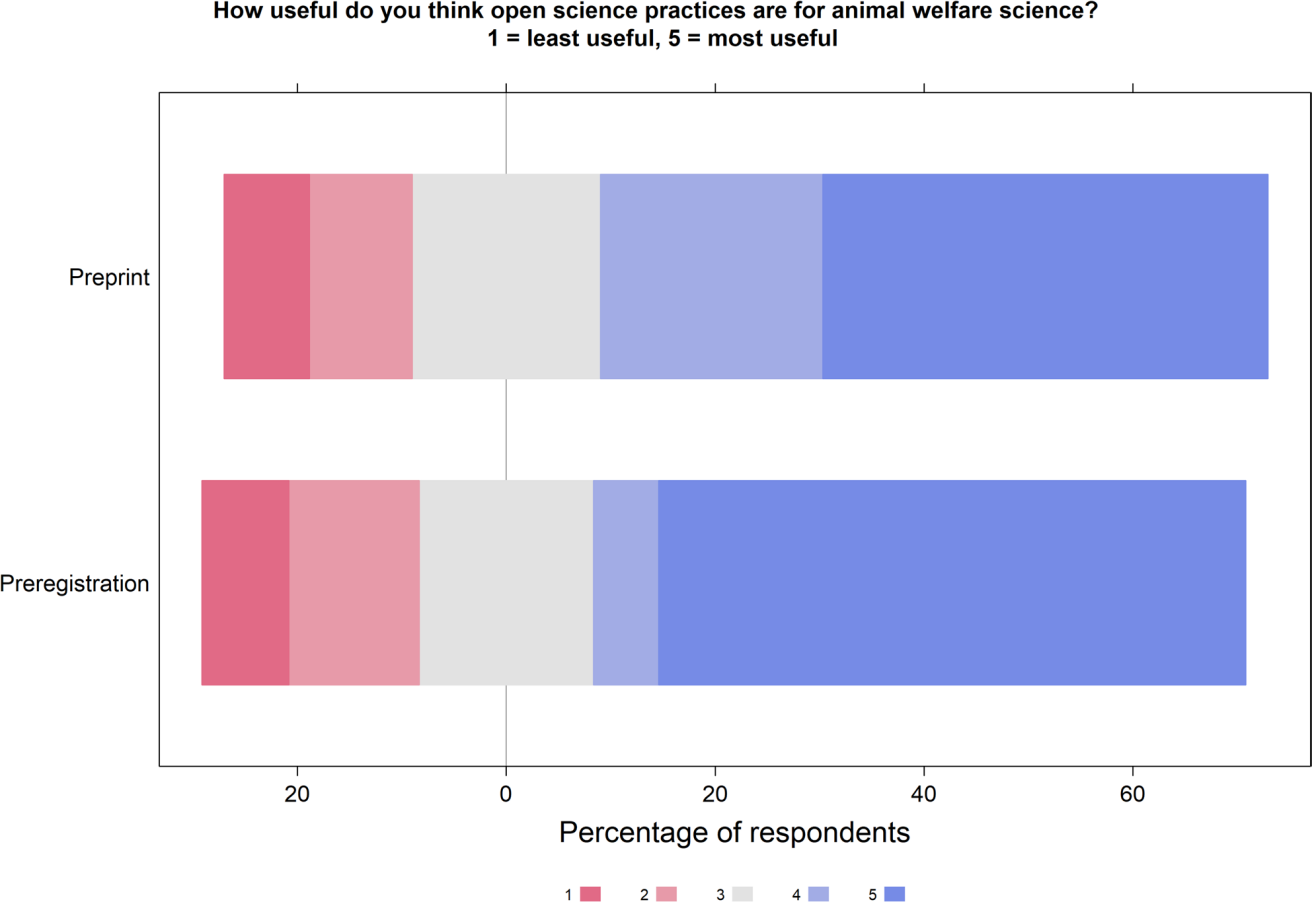
Respondents’ opinions on the usefulness of preprints (*n* = 61) and preregistration (*n* = 48) in animal welfare science. 1 = least useful, 5 = most useful

Of 107 usable responses, 27 people had published a preprint and 80 had not. Of those who had, 12 had done so once, 13 people 2–3 times, one person more than three times, and one person did not answer. Thirteen people published on BioRxiv, five on the Open Science Framework, eight on alternative platforms, and one person did not answer. Of 104 usable responses, nine respondents had previously preregistered a study, leaving 95 who had not. Five had preregistered once, two people 2 to 3 times, and two people did not answer. Three respondents used the format of a Registered Report, two used the Open Science Framework, three respondents used other, non-specified, platforms and two did not respond. The responses explaining reasons for or against a practice, as well as respondents’ plans, are displayed in Fig. 1. Figure 2 displays respondents’ views on how useful they believe both preprints and preregistration are for animal welfare science.

Despite an overall trend for an increase in preprints across disciplines, e.g. [10], the majority (75%) of our respondents had not used preprinting. It is important to note that three respondents reported having not yet published any work or not doing so in their current role. Whether respondents published was not a question we explicitly asked and we therefore highlight that the results may be an underestimate based on respondents publishing status. For those not using preprinting, lack of training was identified as the key barrier. Training was also mentioned as the biggest barrier to preregistration practices, which only 9% of respondents had previously used. These results mirror other disciplines; cardiologists also cite a lack of practical institutional support as a barrier to Open Science uptake [11], and survey results from communication science researchers indicate a perceived lack of training for certain Open Science tools [12].

Though lack of training was the highest self-identified adoption barrier, when asked about other researchers, lack of time, support, incentives and fear of plagiarism were selected at a much higher rate than when respondents answered for themselves. Here, either respondents exaggerate the risks that others may perceive, or they are more likely to select multiple options when they are unsure of others’ motivations. Interestingly, the risk of scooping (i.e., peers “stealing” intellectual content without acknowledging the source) was considered the second most important reason for others not to preprint. Conversely, it was the option with the lowest response when individuals answered regarding their own motivations. Put plainly, Applied Ethologists are not worried about others’ scooping them but believe their peers may be concerned about it.

To encourage the wider use of Open Science practices, it is useful to understand the current rationale of those partaking and those who do not. Of the 25% of respondents who had used preprint servers, the main purpose of doing so was reported as information dissemination. A small percentage had used preregistration, and of those who had, most were motivated by aspects of internal quality control and research integrity.

The early publishing of results which have not undergone peer review is a topic that has received much attention in public health after COVID-19 [13]. Flanagin and colleagues [13] highlight the challenges of sharing non-reviewed results with a wide audience in comparison to sharing with peers who understand discipline-specific nuances in context, methodology and interpretation. Similar challenges may arise in animal welfare science given the strong public interest and controversy over topics under study. Nevertheless, in our study, the practice of preprinting was also seen with a potential to benefit animal welfare science, as 64% (out of 61 respondents) scored either a 4 or 5 (out of 5) for the usefulness of preprints. This notion is also reflected in Nawroth and Krause [7], who argue that Open Science practices can complement strategies to better implement the 3Rs principles in animal research. For example, the low rate of preregistration may contribute to publication bias and to HARKing (i.e., Hypothesizing After the Results are Known), leading to redundant or poorly designed studies, ultimately resulting in unnecessary use of animals and undermining the Reduction principle.

For both preprinting and preregistration, many respondents indicated an interest in adopting these practices in the future, while only a small proportion categorically ruled them out. The results also showed that there is a large proportion of the community surveyed who are unsure whether they want to implement. This may be due to a lack of training and understanding of the benefits and potential pitfalls.

### Open data

Of 106 usable responses, 27% reported always sharing their data, 57% shared sometimes and 15% never shared their data. The limited sharing of research data significantly hinders reproducibility efforts and transparency in science. This is evident in the high percentage of respondents who rarely or only sometimes share their data, despite a substantial interest among researchers in accessing their peers’ data (see Fig. 1) – a discrepancy that indicates that inequity and free-riding hinders reproducibility. Similar patterns appear across disciplines, with a minority of researchers making their data publicly available through methods such as Electronic Supplementary Material (ESM) or repositories [14]. Often, researchers declare that data can be provided upon reasonable request, though many are failing to comply with these commitments [14]. Encouragingly, journal policies mandating data sharing have been shown to enhance compliance, increasing the rate of shared data from 37% to 79% [15, 16].

Only 21% of respondents had heard of the FAIR principles. Whereas 3/16 people who reported never sharing data had heard of FAIR, many who reported sharing data did not know of FAIR. This suggests that many share data in response to journal requirements rather than to optimize access to data, as also supported by our finding that the most common way of sharing data was as a supplementary file. Following the FAIR foundational principles, Findability, Accessibility, Interoperability, and Reusability, is important to maximize the value of data sharing [17].

## Conclusions

In Applied Ethology, there is a clear need for accessible and cross-institutional Open Science training opportunities, which are currently limited and often reliant on broader initiatives or external programs. Understanding the barriers to establishing such training programs is essential for employing structural and educational solutions. Standardized Open Science guidelines and inter-institutional networks should be developed to support the field [18–20]. By fostering a culture of sharing and openness, Applied Ethologists can continue to critically reflect on and implement best practice recommendations to advance their research quality and provide a better implementation of the 3Rs principle.

## Limitations

Surveys offer valuable insights but have methodological limitations. Key concerns include sample representativeness, as participants may not reflect the broader population due to self-selection bias, with respondents often being advocates of Open Science practices. Time constraints may further limit participation. Additionally, question framing can introduce bias, steering answers and distorting opinions [21]. While our survey assessed perceptions of Open Science practices in Applied Ethology research, these perceptions thus may differ from their actual implementation in this field. Indeed, evidence from other fields suggests researchers often underestimate OS practice prevalence in their disciplines [22]. Future research should also aim to standardize questions on specific practices, as our study did not do so for reasons of brevity and practicality. Such standardization will help identify areas where training and interventions can be improved.

## Supplementary Information

Below is the link to the electronic supplementary material.

Supplementary Material 1.

## Data Availability

The anonymised survey data and the analysis code can be accessed here: https://osf.io/dg5e9/?view_only=d5143907ac9c42549553e7ca3de21931.
