# Supplementary material for: Exploring open science in applied ethology: practice and attitudes among researchers
Source: BMC Res Notes. 2026 Feb 6;19:97. doi: 10.1186/s13104-026-07685-x (PMC12947526; doi:10.1186/s13104-026-07685-x)
Supplement: Supplementary file 1 — Supplementary Material 1. [file 13104_2026_7685_MOESM1_ESM.docx]

**Electronic Supplementary Material for “Exploring Open Science in Applied Ethology: Practice and Attitudes among Researchers”**

**Table S1.** Descriptives of the demographic composition of our survey participants.

| Category | Options | No. and % of respondents |
| --- | --- | --- |
| Research Involvement | Currently involved | 106 (95%) |
|  | Involved in the past | 6 (5%) |
| ISAE membership* | Member | 63 (56%) |
|  | Non-member | 49 (44%) |
| Educational status | No PhD - not enrolled in a program | 15 (13%) |
|  | No PhD - enrolled in a program | 36 (32%) |
|  | PhD completed within the last 5 years | 24 (21%) |
|  | PhD completed over 5 years ago | 37 (33%) |
| Age | 18 - 30 | 39 (35%) |
|  | 31 - 45 | 47 (42%) |
|  | 46 - 60 | 20 (18%) |
|  | 61+ | 6 (5%) |

* At the time of the survey, ISAE has listed 649 active members (according to their June 2021 Newsletter

**Table S2.** Overlap of responses: the number of respondents who indicated a similar set of open science practices they engage with.

| **Preprints** | **Preregistration** | **Data_sharing** | **Number of respondents** |
| --- | --- | --- | --- |
| No | No | Sometimes | 42 |
| No | No | Always | 19 |
| No | No | Never | 19 |
| Yes | No | Sometimes | 10 |
| Yes | No | Always | 7 |
| Yes | Yes | Sometimes | 5 |
| No | Yes | Sometimes | 4 |
| Yes | No | Never | 3 |
| Yes | Yes | Always | 2 |
| No | Yes | Always | 1 |
|  |  |  |  |

**Table S3.** Descriptive results of free-text responses representing the relative number of free-text responses to each free-text query.

| **Response category** | **% of free text responses** |
| --- | --- |
| Why do you preprint? | 20.0 |
| Why do you not preprint? | 11.1 |
| Why don't others preprint? | 8.9 |
| How useful are preprints? | 44.6 |
| Why do you preregister? | 27.4 |
| Why do you not preregister? | 0 |
| Why don't others preregister? | 10.7 |
| How useful is preregistration? | 30.4 |

**S4.** Summary of content analysis of free text responses

Data from the free text responses provided further insight, especially for the questions about usefulness of preprints and plans for preregistration the material, for which we present a summary. The full text analyses were manually analysed in MS Excel and categorised into overarching themes. In the following we show illustrative examples of the different categories. Colour codes represent how the cited respondent had answered the question of whether they plan to preprint / preregister, with green for those who plan to, amber for those who do not plan to and yellow for those who do not know.

Considering the **usefulness of preprints** for animal welfare science, respondents highlight the value of speedy publication, which helps moving the field forward, and avoiding overlap in that others learn about the research and its results early.

| **Usefulness** | **Example of response** |
| --- | --- |
| 5 | We could move forward much faster, especially on the newer fronts where the methodology isn't fully sorted |
| 5 | faster access to information, some of which may not be possible to publish in a peer review. I believe in the transparency of science and scientific integrity, and I consider that all information is useful even when the expected results have not been obtained (it can serve to avoid repetitions of work and to take a step forward on the frontier of knowledge) |
| 4 | I think this would be helpful to have access to more current findings so new research can be designed better to extend those findings |

**Preprints** are also seen as an **opportunity for scholarly discussion** around a study, which may improve the paper itself and stimulate interaction among researchers around the topic.

| **Usefulness** | | **Example of response** |
| --- | --- | --- |
| 5 | Pre-prints before even sending to publishers will help to improve the science that is out there as more feedback can be received by researchers. | |
| 5 | If done correctly, preprints can stimulate discussion among peers which will probably improve the quality of the manuscript before submission to a journal, therefore, increase the chances of it being accepted. Furthermore, results will be disseminated much quicker and not behind a paywall, increasing visibility. | |

**Reaching a wider audience** of people who do not have access to paywalled journals is also mentioned.

| **Usefulness** | **Example of response** |
| --- | --- |
| 4 | It will be very useful, as there is a growing interest in animal welfare science especially in the developing countries. |
| 4 | This would give the people within the society research driven information as opposed to opinion blogs |
| 5 | If i cannot read a full article I will not cite it, I don't always have access to journals |

**Reservations** that respondents raise **regarding the value of preprints** have to do with the potentially problematic quality and the limited recognition of papers that have not been peer reviewed, both in terms of using the results and getting credit for the work.

| **Usefulness** | **Example of response** |
| --- | --- |
| 5 | If the scientific community is very active, researchers can discuss actively over a preprint and it is very useful before publication. However, it also contributes to publishing low-quality researches. Unfortunately, publication quality of animal welfare science is not always high and using preprint servers in wrong way is not good. |
| 2 | Information found in pre-prints is not accepted as legitimate support for points in own articles, as this information cannot be cited. Therefore, I think pre-prints are of limited use. |
| 4 | I think if it was recognised by universities and research institutions towards work load models and usable for promotions, then a lot more researchers would consider doing this. |

In the context of **planning to preregister future studies**, respondents refer to how this is relevant for identifying and preventing questionable research practices, increase information sharing in the research community and get relevant feedback on the study plan.

| **Example of response** |
| --- |
| Preregistration helps to identify potentially problematic research practices, such as p-hacking, inappropriate forms of post hoc analysis, publication bias, data dredging and HARKing. |
| It could help cut down on authors retrofitting hypotheses or tweaking analytical methods to get "publishable" results (consciously or unconsciously). It would also help solve the file-drawer problem, in that we would know when studies with "negative" results are performed (helps meta-analyses produce a clearer picture, also helps other researchers know if something has been tried before). Pre-registering might also (I think) actually help make "negative" results more publishable once they are obtained, further reducing incentives to tweak hypotheses or planned analysis methods. |
| It can be very useful, especially for undergraduate and graduate students, to know which research groups and which researchers are doing similar work. It could facilitate the exchange of experiences, methodologies, how to solve research problems, results, etc. |
| Getting expert feedback at the beginning of the study is invaluable. This is the perfect time to actually have an impact in the quality of the study. |

Most **reservations regarding preregistration** have to do with respondents not perceiving this practice as relevant in animal welfare research.

| **Example of response** |
| --- |
| I think it depends on the type of study. For controlled studies in an experimental set-up it will be very useful, for more theoretical studies of animal welfare it won't be useful. |
| Most studies in animal welfare science are exploratory and in these studies there is not only one distinct hypothesis that is tested like in a clinical study. These studies cannot be pre-registered. |
